# Prefrontal-limbic circuit modulation in anxiety disorders: Differential neural effects of psychotherapy versus pharmacotherapy revealed by ALE meta-analysis

**DOI:** 10.1080/19585969.2026.2719522

**Published:** 2026-08-25

**Authors:** Juan Qiu, Junjie Ren, Jin Chang, Limin Huang, Xiaoming Li

**Affiliations:** ^a^ Department of Medical Psychology, School of Mental Health and Psychological Science, Anhui Medical University, Hefei, China; ^b^ Department of Psychiatry, Chaohu Hospital of Anhui Medical University, Hefei, China

**Keywords:** Meta-analysis, anxiety disorders, psychotherapy, pharmacotherapy

## Abstract

**Background:**

Anxiety disorders (ADs) involve dysregulation of prefrontal–limbic circuits, but the neural effects of psychotherapy and pharmacotherapy remain unclear.

**Methods:**

We conducted an activation likelihood estimation (ALE) meta-analysis of task-based fMRI studies reporting pre- and post-treatment whole-brain coordinates in ADs. Separate analyses were performed for psychotherapy and pharmacotherapy. A random-effects synthesis assessed changes in primary anxiety symptoms where extractable data were available.

**Results:**

27 studies involving 561 patients were included. Psychotherapy was associated mainly with decreased activation in prefrontal and striatal regions, including the medial, middle, and inferior frontal gyri and caudate nucleus, whereas pharmacotherapy showed more focal decreases in the right uncus. Subgroup analyses suggested heterogeneity across therapies, diagnoses, and tasks. 9 independent active-treatment cohorts (179 participants) showed a large pooled symptom reduction (Hedges’ g = 1.44, 95% CI 0.99–1.90; I² = 75.1%).

**Conclusions:**

Psychotherapy and pharmacotherapy may engage partly distinct neural pathways in ADs, with broader prefrontal–striatal modulation after psychotherapy and more focal limbic effects after pharmacotherapy. Findings remain exploratory given the limited and heterogeneous evidence base.

## Introduction

Anxiety is an adaptive response that promotes vigilance and coping when a threat is proportionate and time limited; it becomes pathological when fear or worry is excessive, persistent, difficult to regulate, and associated with avoidance, clinically significant distress, or functional impairment (Stein et al. 2014). Anxiety disorders (ADs) are among the most prevalent mental disorders worldwide and are characterised by excessive fear and/or anxiety, often accompanied by persistent worry and marked functional impairment (Stein et al. 2014). Global Burden of Disease analyses document their substantial worldwide burden; in 2021, an estimated 359.21 million people were affected, corresponding to an age-standardised prevalence of 4421.87 per 100,000 population (Zhou et al. 2025, 3; COVID-19 Mental Disorders Collaborators 2021). The present review focuses on panic disorder (PD), social anxiety disorder (SAD), and specific phobia (SP). Their aetiology is multifactorial, reflecting interactions among genetic vulnerability, psychosocial stressors, and environmental influences, and current clinical management primarily relies on psychotherapy and pharmacotherapy.

At the neurobiological level, ADs are associated with interacting inhibitory, excitatory, monoaminergic, and neuroendocrine systems rather than a single transmitter abnormality. Alterations involving GABA and glutamate, serotonergic, noradrenergic, and dopaminergic modulation, and hypothalamic-pituitary-adrenal (HPA)-axis regulation have all been investigated, although none currently constitutes a disorder-specific diagnostic biomarker (Bandelow et al. 2017). A large genome-wide association meta-analysis identified 58 independent risk variants and 66 genes with robust biological support, highlighted GABAergic signalling, and demonstrated substantial genetic correlation between anxiety disorders and depression and other internalising phenotypes (Strom et al. 2026). Candidate-gene research has also examined COMT and MAOA because these enzymes influence catecholamine and monoamine metabolism; however, reported associations remain heterogeneous and should be interpreted cautiously (Faludi et al. 2012). The shared genetic, monoaminergic, stress-response, and circuit-level features may partly account for the frequent clinical interplay between anxiety and mood disorders (Faludi et al. 2012; Bandelow et al. 2017; Strom et al. 2026).

Evidence-based research shows that psychological interventions such as Cognitive-Behavioural Therapy (CBT), Mindfulness-Based Stress Reduction (MBSR), psychodynamic therapy, and interpersonal therapy are effective (Leichsenring and Leibing 2003; Cuijpers et al. 2016; Hoge et al. 2023), with CBT recommended as the first-line treatment due to its structured nature (Hofmann and Smits 2008). However, approximately 47% of patients are resistant to CBT (Loerinc et al. 2015), highlighting the need to explore its mechanisms to optimise efficacy. In terms of pharmacotherapy, Selective Serotonin Reuptake Inhibitors (SSRIs) and Serotonin-Norepinephrine Reuptake Inhibitors (SNRIs) are first-line drugs (Bandelow et al. 2012), but a meta-analysis of 37,333 patients showed their effect sizes (*g* = 0.38) are only marginally higher than those of psychotherapies (*g* = 0.31) (Bandelow et al. 2015), and they are often accompanied by adverse effects such as gastrointestinal reactions and sexual dysfunction (DeMartini et al. 2019). These limitations have prompted research into the neural mechanisms underlying treatment responses involving prefrontal–limbic regulation circuits, amygdala reactivity, and fronto-striatal control systems (Etkin and Wager 2007). SSRIs primarily inhibit the serotonin transporter, whereas SNRIs inhibit both serotonin and norepinephrine transporters; the relative contribution of the two systems varies across agents and doses, although comparative evidence indicates only small overall differences in efficacy and acceptability between the classes (Gosmann et al. 2021). Oxytocin was included in the search because experimental anxiety studies have examined it as a putative neuromodulator of amygdala-centered social-threat and emotion-regulation circuits; current clinical evidence remains preliminary and inconclusive (De Cagna et al. 2019).

Advances in functional magnetic resonance imaging (fMRI) have provided an important avenue for investigating treatment-related neural changes in ADs. Task-based fMRI studies indicate that activity within prefrontal–limbic circuits is associated with treatment response and symptom improvement. For example, prefrontal–amygdala connectivity has been shown to predict response to CBT in SAD (Klumpp et al. 2014), while prefrontal activation during threat processing has been linked to treatment response in anxious youth, regardless of whether they receive pharmacotherapy or psychotherapy (Kujawa et al. 2016). However, despite the growing number of neuroimaging studies, existing task-based meta-analyses have largely focused on a single treatment modality, without directly comparing the neural effects of psychotherapy and pharmacotherapy within a unified quantitative framework. Psychotherapy is commonly conceptualised as modifying learned threat appraisal and strengthening regulatory processes through exposure, cognitive reappraisal, or attentional training, whereas pharmacotherapy may alter emotional-processing biases through serotonergic and/or noradrenergic mechanisms. Previous neuroimaging syntheses, including those by Messina et al. (2013), Quidé et al. (2012), and Marwood et al. (2018), have reported treatment-related changes across prefrontal-limbic systems but also emphasised substantial heterogeneity, supporting the present modality-specific ALE synthesis. Combined psychotherapy and pharmacotherapy are also used clinically and may improve outcomes relative to pharmacotherapy alone in some anxiety disorders (Cuijpers et al. 2014); however, combined interventions were excluded from the present synthesis to isolate modality-specific neural effects and avoid attributing an observed change to either component when both were delivered concurrently.

Activation likelihood estimation (ALE) meta-analysis offers a rigorous approach to address these limitations by quantitatively assessing the spatial convergence of activation across independent neuroimaging studies while accounting for sample size and spatial uncertainty (Turkeltaub et al. 2002). By synthesising task-based fMRI findings at the whole-brain level, ALE enables identification of robust and reproducible neural patterns associated with specific treatment modalities (Müller et al. 2018). Therefore, the present study employed ALE meta-analysis to systematically examine treatment-related brain activation changes following psychotherapy and pharmacotherapy in ADs. This study uses ALE to systematically integrate neuroimaging evidence from psychotherapy and pharmacotherapy, aiming to answer 2 core questions: (1) What are the characteristic neural activation patterns associated with psychotherapy and pharmacotherapy, respectively? (2) To what extent do these treatments engage overlapping or distinct brain circuits? Given the limited and heterogeneous evidence base, the analyses focused on modality-specific comparisons rather than pooled conjunctions. This work aims to provide a more reliable summary of task-based neural changes underlying treatment effects in ADs, contributing to future hypothesis generation and precision treatment frameworks.

## Methods

### Literature search

A systematic search was conducted for studies published between January 1, 2000, and December 31, 2025. Seven databases were searched: PubMed, ClinicalTrials.gov, Embase, Web of Science, Cochrane Library, Ovid Medline, and PsycINFO. Search terms were grouped into 4 categories: Group 1, ‘anxiety disorders’, ‘generalised anxiety disorder’, ‘panic disorder’, ‘social anxiety disorder’, and ‘specific phobia’; Group 2, ‘psychotherapy’, ‘cognitive behaviour therapy’, ‘mindfulness-based stress reduction’, ‘exposure therapy’, and ‘attention bias modification’; Group 3, ‘pharmacotherapy’, ‘paroxetine’, ‘sertraline’, ‘oxytocin’, ‘citalopram’, and ‘fluoxetine’; and Group 4, ‘functional magnetic resonance imaging’. Terms within each group were combined with OR, and the four groups were combined with AND. The complete PubMed Boolean search strategy is provided in Supplementary Table S1.

Additionally, the reference lists of three relevant neuroimaging reviews and meta-analyses—Messina et al. (2013), Quidé et al. (2012), and Marwood et al. (2018)—were searched manually to identify potentially eligible studies. Corresponding authors of included studies were contacted to clarify ambiguous data (e.g., unreported coordinates). This meta-analysis adhered strictly to the PRISMA guidelines and was prospectively registered with PROSPERO (CRD420251127853).

### Inclusion and exclusion criteria

Inclusion Criteria: (1) Participants: Patients with a confirmed diagnosis of ADs (PD, SAD, or SP) *via* DSM-IV, DSM-5, or ICD-11; age, gender, and recruitment source were unrestricted; (2) Treatment Modality: Single-modality intervention (psychotherapy or pharmacotherapy) with a duration of ≥ 3 weeks; (3) Sample Size: ≥ 5 participants per treatment group (to reduce bias from very small samples); (4) Neuroimaging: task-based fMRI studies reporting pre- and post-treatment changes in brain activation. Studies reporting whole-brain analyses were eligible for inclusion in the main ALE meta-analysis, whereas studies reporting only region-of-interest (ROI)-based results were not included in the primary ALE analyses and were handled separately in supplementary or descriptive analyses; (5) Reporting Standard: Pre- and post-treatment changes in brain regions reported using stereotactic coordinates (x, y, z) in MNI or Talairach space; and (6) Assessment Timing: Neuroimaging at baseline (pre-treatment) and end of treatment (post-treatment).

Exclusion Criteria: (1) Patients with significant comorbidities (e.g., schizophrenia, bipolar disorder) or recent (≤ 3 months) psychotropic medication use (to avoid confounding neuroplasticity); (2) Case studies, reviews, or meta-analyses; (3) Combined treatments (defined as concurrent use of psychotherapy and pharmacotherapy at any point during the intervention period); (4) Studies lacking coordinate data for brain activation changes; (5) Concurrent treatments (e.g., neuromodulation alongside psychotherapy/pharmacotherapy).

The eligibility framework was specified according to PICO: Population, patients with PD, SAD, or SP; Intervention, single-modality psychotherapy or pharmacotherapy lasting at least 3 weeks; Comparator, the within-patient pre-treatment condition (with a healthy or clinical comparison group where reported, but not required for eligibility); and Outcomes, treatment-related changes in task-fMRI activation reported as stereotactic whole-brain coordinates for the primary ALE analyses.

### Data extraction

Data extraction was performed independently by 2 researchers (J.Q. and J.R.) on January 20, 2026, and subsequently reviewed for accuracy and consistency by the corresponding author. Extracted variables included demographics (sample size, age, gender ratio); treatment details (modality, duration, sessions); fMRI parameters (task paradigm, scanning resolution, template: MNI/Talairach); and neuroimaging results (stereotactic coordinates of treatment-related activation changes and classification of findings as whole-brain or region-of-interest-based; ROI-derived results were not included in the ALE analyses). For the behavioural synthesis, the reviewers also extracted the primary anxiety measure, the analytic sample size, pre-treatment and post-treatment means and standard deviations, and the standard deviation of change or other paired-dispersion information when reported.

To comply with the methodological assumptions of ALE, only activation coordinates derived from whole-brain analyses were included in the primary ALE meta-analyses. Coordinates reported exclusively from ROI analyses were not included in the main ALE analyses and were handled separately in supplementary or descriptive analyses.

When both whole-brain and ROI-based results were reported within the same study, only the whole-brain coordinates were extracted and entered into the primary ALE analyses. This approach was adopted to avoid potential inflation of spatial convergence and distortion of the null distribution associated with spatially constrained ROI-derived coordinates, in accordance with established BrainMap/GingerALE methodological guidelines (Eickhoff et al. 2012).

### Meta-analysis

The ALE meta-analysis approach, first introduced by Turkeltaub et al. (2002), involves estimating the probability of voxel activation under specific experimental conditions and integrating these estimates. To examine coordinates provided in either Talairach or MNI space, we utilised GingerALE 3.0.2—a tool designed for ALE meta-analysis within the BrainMap environment (Eickhoff et al. 2009). Treatment-related changes were identified by extracting three-dimensional coordinates from within-group, pre-versus post-treatment contrasts. All coordinates originally reported in Talairach space were converted to MNI space to maintain consistency, as our meta-analysis adhered to the standard MNI reference system (Eickhoff et al. 2009). Following common practices in ALE meta-analyses under conditions of limited sample size and high heterogeneity, we applied a voxel-wise threshold of *p* < 0.001 (uncorrected) combined with a minimum cluster extent of 250 mm³ (Eickhoff et al. 2012). Because this threshold does not control the family-wise error rate or false discovery rate, the maps were treated as exploratory and non-confirmatory throughout; the thresholding specification was reported explicitly in the tables and figure legends (Eickhoff et al. 2012; Müller et al. 2018). To investigate brain-activation changes in patients with ADs following psychotherapy or pharmacotherapy, we conducted a functional-imaging meta-analysis. We examined task-related changes in brain activity associated with both psychotherapy and medication, identifying consistent post-treatment alterations across all eligible functional-imaging studies. Separate ALE analyses were performed for psychotherapy and pharmacotherapy to identify modality-specific activation likelihoods, without conducting pooled conjunction analyses due to limited sample overlap. We carried out subgroup analyses stratified by treatment type and task paradigm. ALE outcome maps and corresponding cluster reports delineated the locations and probabilities of activated brain regions. Finally, we used Mango software to overlay significant coordinates onto standard brain templates for visualisation of the activation clusters.

### Behavioural and demographic synthesis

Where a primary anxiety symptom measure provided pre-treatment and post-treatment means and dispersion data, within-group standardised mean changes were calculated as Hedges’ g, with positive values denoting symptom reduction. A random-effects model with restricted maximum likelihood estimation was used. When the standard deviation of the change score was not reported, it was derived as SD_change = sqrt(SD_pre^2^ + SD_post^2^ – 2r × SD_pre × SD_post). Because the within-person correlation is required for the sampling variance of a standardised mean change but is often unreported, *r* = 0.50 was used for the primary analysis and *r* = 0.30 and *r* = 0.70 were examined in sensitivity analyses (Morris 2000; Cuijpers et al. 2017). One primary anxiety measure was selected per independent active-treatment cohort. Study-level calculations and REML pooling were implemented in Python 3.12.13 using NumPy 2.3.5. Demographic characteristics were summarised from the originally extracted study-level data; formal pooling of age and sex was not undertaken because reporting was incomplete and several publications drew on overlapping cohorts. Cohort-level inputs, effect sizes, and standard errors are provided in Supplementary Table S10; the forest plot and sensitivity analysis are shown in Supplementary Figures S6 and S7.

### Quality assessment of included studies

Because no standardised checklist exists for evaluating the methodological quality of task-based functional neuroimaging studies, we adopted a structured evaluation approach based on previously published fMRI meta-analyses (Zhang et al. 2024).

The checklist contained 10 subitems grouped into 3 domains: participants; image acquisition and analysis; and results and conclusions (see Supplementary Table S2). Each subitem was rated as 0 (not met), 0.5 (partially met), or 1 (fully met), yielding a total possible score of 0–10 for each study. According to the total score, studies with higher total scores were considered to have better methodological quality. 2 reviewers independently performed the assessment, and any discrepancies were resolved through consensus with a senior author. Detailed scores for individual studies are provided in Supplementary Table S3.

## Results

### Basic description of the included literature

**Figure 1.**
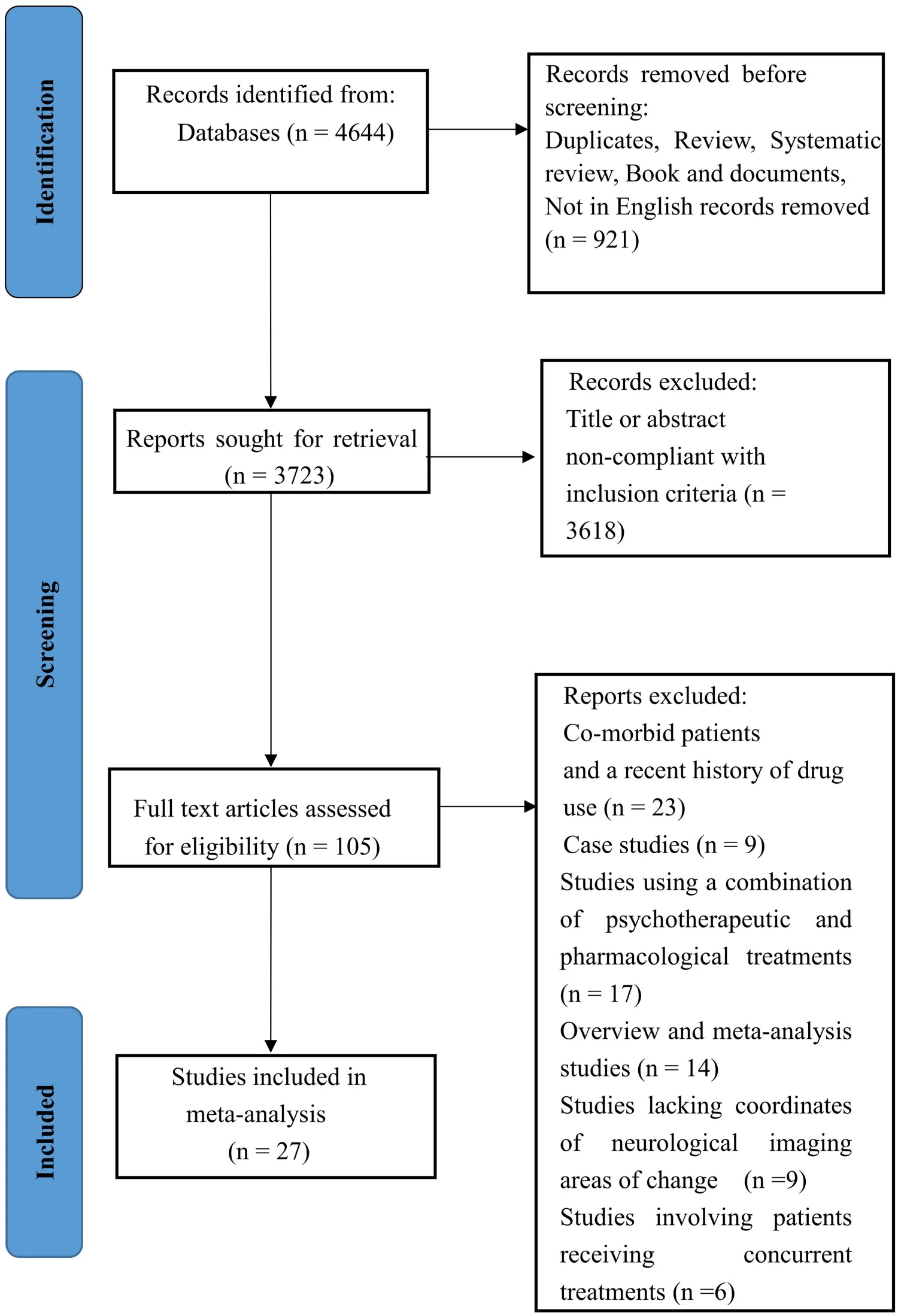
Preferred Reporting Items for Systematic Reviews and Meta-Analyses (PRISMA) flow diagram of search strategy.

The literature search identified 4644 studies. After title and abstract screening, 105 full-text articles were assessed for eligibility, and 27 studies (Furmark et al. 2002; Hoehn-Saric et al. 2004; Kilts et al. 2006; Straube et al. 2006; Schienle et al. 2007; Goldin et al. 2009; Sim et al. 2010; Schneier et al. 2011; Goldin et al. 2012; Hauner et al. 2012; Hölzel et al. 2013; Kircher et al. 2013; Klumpp et al. 2013; Lueken et al. 2013; Månsson et al. 2013; Phan et al. 2013; Klumpp et al., 2014; Lipka et al. 2014; Klumpp et al. 2016; Faria et al. 2017; Brown et al. 2019; Kunas et al. 2019; Bomyea et al. 2020; Álvarez-Pérez et al. 2021; Hur et al. 2021; Kim et al. 2022; Azriel et al. 2024) met the inclusion criteria (Figure 1). These 27 studies included a total of 561 patients with ADs, all diagnosed using DSM (IV/5) or ICD-11 criteria. Detailed characteristics of the included studies are provided in Table 1.

**Table 1.** Characteristics of psychotherapy inclusion in the literature.

Study*	N (F)		Age (SAD)		Therapy	Disorder	Task	Diagnosis	Coordinate Space	Sessions (weeks)
	HC	ADs	HC	ADs						
Psychotherapy										
Brown et al. (2019)	14(8)	17(17)	29.1(7.4)	27.3(6.0)	CBT	SAD	Speech performance task	DSM-IV	MNI	12 weeks
Goldin et al. (2009)	–	16(9)	–	35.2(11.9)	MBSR	SAD	Self-referential encoding task	DSM-IV	Talairach	8 weeks
Goldin et al. (2012)	–	24(19)	–	32.87(8.83)	MBSR	SAD	Self-referential encoding task	DSM-IV	Talairach	8 weeks
Hauner et al. (2012)	–	12(9)	–	23(4.5)	ET	SP	Responses to phobogenic stimuli	DSM-IV	Talairach	12 weeks
Hölzel et al. (2013)	–	15(9)	–	38.5(13.3)	MBSR	SAD	Emotional Face Recognition Task	DSM-IV	MNI	8 weeks
Klumpp et al. (2013)	14(8)	14(9)	23.29(5.44)	28.07(8.62)	CBT	SAD	Emotional Face Recognition Task	DSM-IV	MNI	12weeks
Klumpp et al. (2016)	–	32(24)	–	25.4(5.1)	CBT	SAD	Visual Selective Attention Task with Face Distractors	DSM-IV	MNI	12 weeks
Klumpp et al. (2014)	–	21(15)	–	24.9(6.3)	CBT	SAD	Emotional Face Recognition Task	DSM-IV	MNI	12 weeks
Kunas et al. (2019)	–	27(18)	–	34.51(9.51)	CBT	PD	Fear Conditioning Tasks	DSM-IV	MNI	12weeks
Lipka et al. (2014)	16(16)	14(14)	23.88(3.65)	25(3.7)	CBT	SP	View Spider Pictures	DSM-IV	Talairach	2 weeks
Lueken et al. (2013)	–	49(33)	–	35.27(10.43)	CBT	PD	Fear Conditioning Tasks	DSM-IV	MNI	12weeks
Månsson et al. (2013)	–	13(11)	–	32.08(10.9)	ABM	SAD	Emotional Face Recognition Task	DSM-IV	MNI	9 weeks
Månsson et al. (2013)	–	13(11)	32.08(10.9)	32.46(8.6)	CBT	SAD	Emotional Face Recognition Task	DSM-IV	MNI	9 weeks
Kircher et al. (2013)	42(29)	42(29)	35.42	34.33	CBT	PD	Conditional response	DSM-IV	MNI	12 sessions
Álvarez-Pérez et al. (2021)	–	14(13)	–	29.64	CBT	SP	View Spider Pictures	DSM-IV	MNI	3 weeks
Hur et al. (2021)	22(10)	25(10)	23.95(3.5)	23.04(3.35)	VR	SAD	Self-referential emotional processing	DSM-IV	MNI	6 sessions
Furmark et al. (2002)	–	6(3)	–	35.2(7.3)	CBT	SP	Public Speaking Task	DSM-IV	Talairach	4 weeks
Kim et al. (2022)	20(13)	21(13)	23.5(2.2)	23.6(2.8)	VRS	SAD	Speech Task	DSM-IV	MNI	3 weeks
Azriel et al. (2024)	30(12)	29(11)	29.66(7.06)	29.59(7.03)	ABM	SAD	Emotional Face Recognition Task	DSM-IV	MNI	12 weeks
Bomyea et al. (2020)	–	30(22)	–	36.6(10.4)	CBT	SAD	Reapprasial Task	DSM-IV	Talairach	12 weeks
Schienle et al. (2007)	25(25)	14(14)	24.6(6.3)	27.2(9.2)	CBT	SP	View Spider Pictures	DSM-IV	MNI	4weeks
Straube et al. (2006)	14(14)	13(13)	22.07(1.98)	21.92(2.02)	CBT	SP	View Spider Pictures	DSM-IV	Talairach	4 weeks
Pharmacotherapy										
Schneier et al. (2011)	16(10)	16(10)	30.3(9.7)	29.8(9.0)	Paroxetin	SAD	Emotional Face Recognition Task	DSM-IV	MNI	8 weeks
Furmark et al. (2002)	–	6(3)	–	35.2(7.3)	Citalopram	SP	Public Speaking Task	DSM-IV	Talairach	4 weeks
Phan et al. (2013)	19(9)	21(13)	26.95(8.11)	5.91(5.50)	Sertraline	SP	Emotional Face Recognition Task	DSM-IV	MNI	12 weeks
Sim et al. (2010)	–	5(2)	–	42.6(7.0)	Paroxetin	PD	Public Speaking Task	DSM-IV	Talairach	12 weeks
Kilts et al. (2006)	6(2)	12(5)	32.4(5.8)	38(8.1)	Nefazodone	SAD	Script-Guided Social Anxiety Imagery	DSM-IV	Talairach	8 weeks
Faria et al. (2017)	–	47(18)	–	31.8(10.2)	Escitalopram	SAD		DSM-IV	MNI	9 weeks
Hoehn-Saric et al. (2004)	–	6(3)	–	36	Citalopram	SAD	Emotional Statements Recognition Task	DSM-IV	MNI	7 weeks

*Studies labelled with first author and publication year; ABM: Attentional Bias Modification Therapy; ADs: anxiety disorders; CBT: Cognitive Behavioural Therapy; ET: Exposure Therapy; GSAD: Generalised Anxiety Disorder; HC: Healthy Controls; MBSR: Mindfulness-Based Stress Reduction; PD: Panic Disorder; SAD: Social Anxiety Disorder; SP: Spider Phobia.

### Results of ALE meta-analysis

#### General overview of treatment-related brain activation changes

A total of 27 task-based fMRI studies comprising 561 participants were included in the ALE meta-analysis. Primary analyses were conducted separately for psychotherapy (21 studies) and pharmacotherapy (7 studies), with 1 overlapping study contributing data to both treatment categories. Given the limited number of eligible studies and substantial heterogeneity across task paradigms and treatment protocols, no conjunction or overlap analyses were performed between treatment modalities.

All results reported below were derived from whole-brain voxel-wise analyses and should be interpreted as exploratory. Across analyses, treatment-related activation changes were predominantly characterised by decreases within prefrontal, limbic, and striatal regions, suggesting the involvement of prefrontal-limbic and prefrontal-striatal regulatory circuits in interventions for ADs.

#### Brain region activation in patients with ADs receiving psychotherapy

21 studies (Furmark et al. 2002; Straube et al. 2006; Schienle et al. 2007; Goldin et al. 2009, 2012; Hauner et al. 2012; Hölzel et al. 2013; Kircher et al. 2013; Klumpp et al. 2013; Lueken et al. 2013; Månsson et al. 2013; Klumpp et al., 2014; Lipka et al. 2014; Klumpp et al. 2016; Brown et al. 2019; Kunas et al. 2019; Bomyea et al. 2020; Álvarez-Pérez et al. 2021; Hur et al. 2021; Kim et al. 2022; Azriel et al. 2024) examining psychotherapy were included in the primary ALE analysis. Exploratory ALE results indicated that psychotherapy-related neural changes were exclusively characterised by decreases in activation, with no clusters showing increased activation.

**Figure 2.**
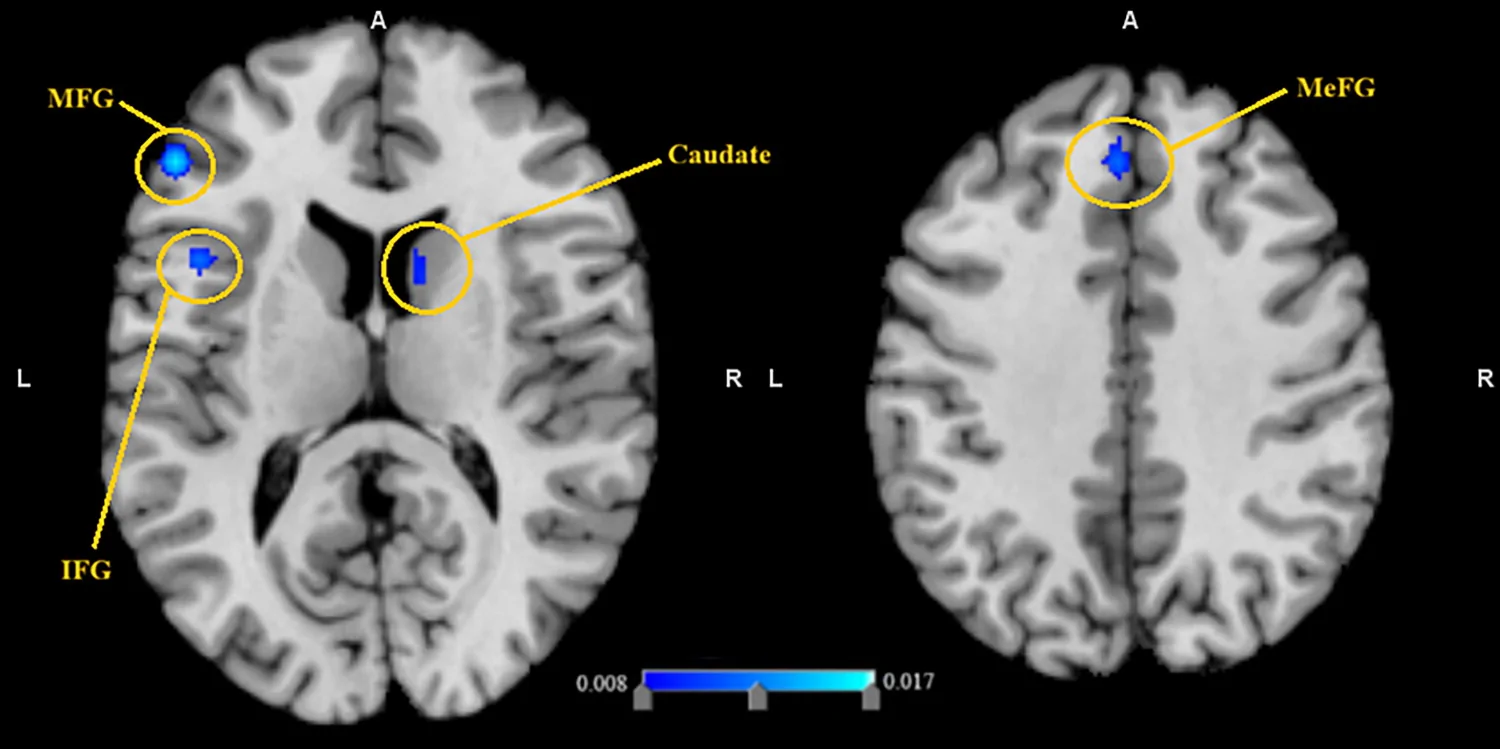
ALE meta-analytic activation cluster results plot for patients with ADs receiving psychotherapy. Uncorrected *p* < 0.001, mni.cluster size = 250mm^3^. The brain regions activated in figure reached a significant activation level, with increased activity areas in red and decreased activity areas in blue. ACG: Anterior Cingulate Gyrus; IFG: Inferior Frontal Gyrus; MFG: Middle Frontal Gyrus; MeFG: Medial Frontal Gyrus.

Specifically, decreased activation was observed in 4 clusters. These clusters were located in the left medial frontal gyrus (MeFG), left middle frontal gyrus (MFG), left inferior frontal gyrus (IFG), and the right caudate nucleus (Figure 2; Table 2). All identified clusters exceeded the predefined minimum cluster size threshold.

**Table 2.** Results of ALE meta-analysis activation clusters in patients with ADs treated with psychotherapy.

Hemisphere	BA	Effect lobe	Effect gyrus	Center coordinates			Volume/mm³	ALE value*
				X	Y	Z		
Decrease:								
Left	8	Frontal Lobe	MeFG	−2	36	38	656	0.012
Left	46	Frontal Lobe	MFG	−50	36	10	544	0.018
Left	44	Frontal Lobe	IFG	−50	10	14	432	0.013
Right		Sub-lobar	Caudate	12	8	12	272	0.010

*The results are in scientific notation and are actually ALE value x 10^−2^. The peak coordinates were in the MNI system. Uncorrected *p* < 0.001, mni.cluster size = 250mm3. ACG: Anterior Cingulate Gyrus; ALE: Activation Likelihood Estimation; BA: Brodmann Area; IFG: Inferior Frontal Gyrus; MFG: Middle Frontal Gyrus; MeFG: Medial Frontal Gyrus.

Overall, these findings suggest that psychotherapy for ADs is associated with reduced activation in prefrontal and striatal regions implicated in cognitive control, emotional regulation, and habitual response processing. Given the uncorrected statistical threshold and the limited number of eligible studies, these results should be interpreted as exploratory.

In the subgroup analysis restricted to studies employing CBT, exploratory ALE results revealed both increased and decreased activation patterns.

Increased activation was observed in 4 clusters, including the right parahippocampal gyrus (PG), bilateral anterior cingulate gyrus (ACG), and the right IFG. In contrast, decreased activation was identified in 3 clusters located in the left MFG, left IFG, and the right caudate nucleus (Supplementary Figure S1 and Table S4).

To further examine diagnosis-specific neural patterns associated with psychotherapy, exploratory ALE subgroup analyses were conducted according to diagnostic category, including SAD, PD, and SP. All results were derived from whole-brain voxel-wise analyses and should be interpreted as exploratory. In patients with SAD, exploratory ALE analysis revealed a single cluster of decreased activation located in the left MeFG. No clusters showing increased activation were identified (Supplementary Table S5 and Figure S2).

In the PD subgroup, exploratory ALE results indicate decreased activation in 2 clusters located in the left MFG and the left insula. No regions showing increased activation were observed (Supplementary Table S6 and Figure S3).

**Figure 3.**
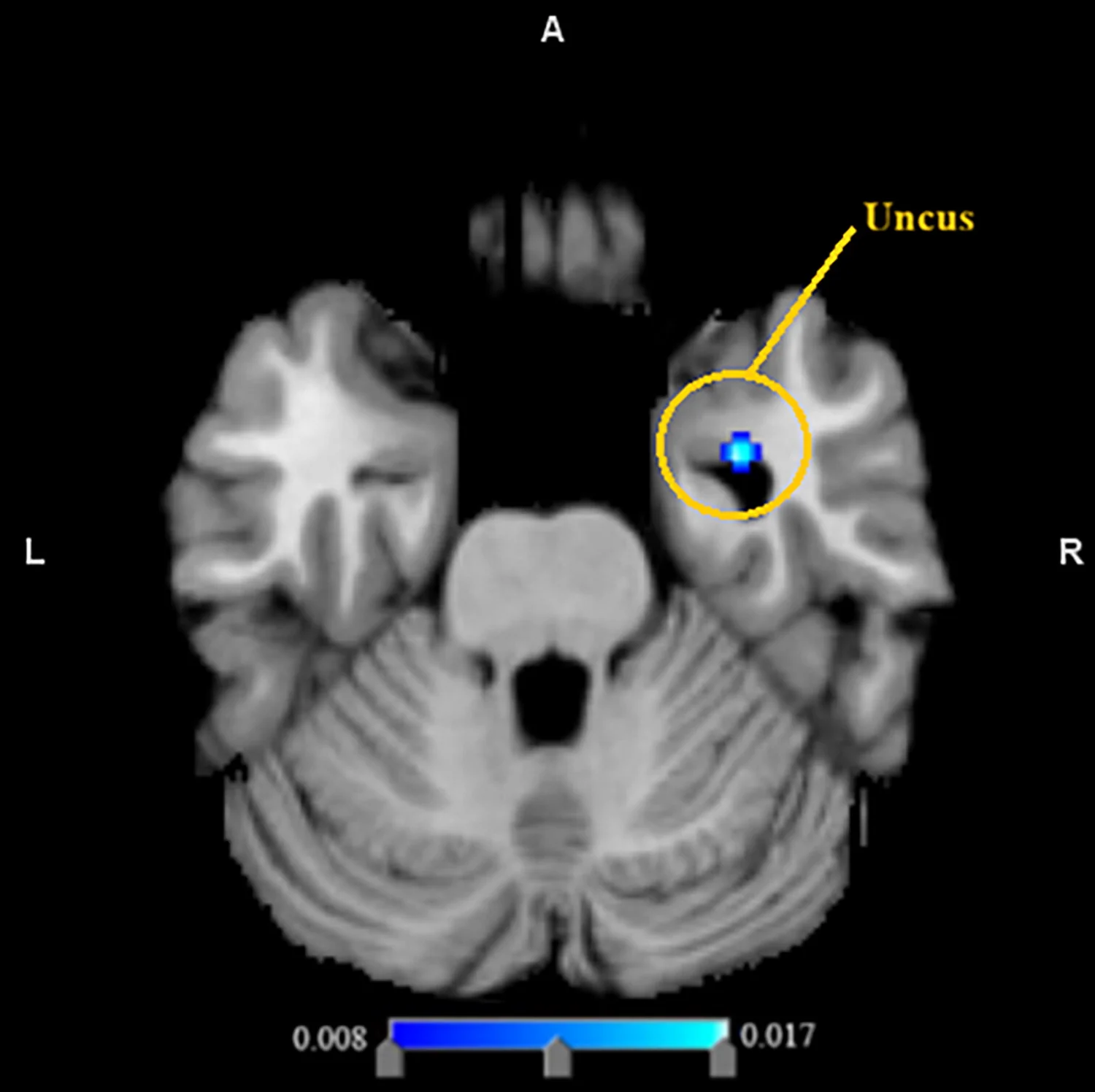
ALE meta-analysis of activation cluster results in patients with ADs treated with medication. Uncorrected *p* < 0.001, mni.cluster size = 250mm^3^. The brain regions activated in figure. reached a significant activation level, with increased activity areas in red and decreased activity areas in blue.

In contrast, exploratory ALE analysis in patients with SP revealed increased activation across 4 clusters. These clusters were located in the left ACG, Middle Occipital Gyrus (MOG), right Frontal Gyrus (FG), and right IFG. No clusters showing decreased activation were identified in this subgroup (Supplementary Table S7 and Figure S4).

In the emotional faces task subgroup, increased activation was observed in the right IFG, left ACG, and right Precentral Gyrus (PreCG) (Supplementary Table S8 and Figure S5).

Overall, psychotherapy-related activation changes differed across diagnostic subgroups. SAD and PD were predominantly characterised by decreased activation in prefrontal and insular regions, whereas SP was associated with increased activation in limbic, occipital, and frontal regions. Given the limited number of studies within each subgroup and the use of an uncorrected statistical threshold, these findings should be interpreted cautiously.

#### Brain region activation in patients with ADs treated with pharmacotherapy

7 studies (Furmark et al. 2002; Hoehn-Saric et al. 2004; Kilts et al. 2006; Sim et al. 2010; Schneier et al. 2011; Phan et al. 2013; Faria et al. 2017) examining pharmacotherapy were included in the ALE meta-analysis. Exploratory ALE analysis revealed a single cluster of decreased activation located in the right uncus within the limbic lobe (Figure 3; Table 3). No clusters showing increased activation were identified.

**Table 3.** Results of ALE meta-analysis activation clusters in patients with ADs treated with pharmacotherapy.

Hemisphere	BA	Effect lobe	Effect gyrus	Center coordinates			Volume/mm³	ALE value*
				X	Y	Z		
Decrease:								
Right	6	Limbic lobe	Uncus	32	−2	−28	256	0.009

*The results are in scientific notation and are actually ALE value x 10^−2^. The peak coordinates were in the MNI system. Uncorrected *p* < 0.001, mni.cluster size = 250mm3. ALE: Activation Likelihood Estimation; BA: Brodmann Area.

Overall, pharmacotherapy-related neural changes were limited in spatial extent. Given the small number of eligible studies and the use of an uncorrected statistical threshold, these findings should be interpreted as exploratory.

### Behavioural and demographic synthesis

Nine independent active-treatment cohorts (179 participants) provided sufficient data for quantitative synthesis of anxiety symptom change. The primary random-effects estimate showed a large pooled standardised mean reduction (Hedges’ *g* = 1.44, 95% CI 0.99–1.90), with substantial heterogeneity (I^2^ = 75.1%; τ^2^ = 0.37; *Q* = 32.16; Supplementary Figure S6). Sensitivity analyses produced *g* = 1.29 (95% CI 0.86–1.71; I^2^ = 74.8%) when *r* = 0.30 and *g* = 1.72 (95% CI 1.19–2.24; I^2^ = 76.3%) when *r* = 0.70, indicating that the direction of improvement was stable (Supplementary Figure S7). All cohort-level means, standard deviations, and analytic sample sizes were checked against the source reports. The behavioural denominator represents participants contributing paired outcome data and may differ from the baseline imaging sample in Table 1 because of attrition or multiple active-treatment arms; these differences are reconciled in the note to Supplementary Table S10. The 27 included studies comprised 561 patients; study-level age and sex distributions are presented in Table 1, but were not formally pooled because of incomplete reporting and overlapping cohorts.

### Quality assessment results

All included studies demonstrated moderate-to-high methodological quality, with total scores ranging from 8.0 to 10.0 (mean ± SD = 9.2 ± 0.6). No study was excluded on the basis of quality assessment. Inter-rater agreement between the 2 reviewers was high. A summary of scoring criteria and individual study ratings is provided in Supplementary Tables S2 and S3.

Excluding lower-quality studies did not materially change the results, supporting the robustness of the findings.

### Descriptive synthesis of ROI-based findings

Although ROI-based results were not included in the present ALE analyses due to well-established methodological constraints, several of the included fMRI studies reported ROI-level activation changes following treatment. These findings are summarised descriptively in Supplementary Table S9 to provide complementary contextual information and should be interpreted independently from the whole-brain ALE results.

Across studies employing CBT, ROI analyses most frequently focused on limbic and paralimbic regions implicated in fear processing and emotion regulation, including the amygdala, ACG, insula, PG, and thalamus. In patients with SP, CBT was associated with reduced activation within these regions, suggesting attenuation of heightened threat-related responses at the regional level. Similarly, decreased amygdala activation following CBT was reported in SAD, consistent with prior observations of reduced limbic reactivity during affective processing tasks.

In contrast, 1 study employing ABM reported increased amygdala activation following treatment, highlighting potential differences in neural engagement across psychological intervention strategies. Importantly, these ROI-based observations were derived from hypothesis-driven regional analyses and do not constitute evidence of spatial convergence. Rather, they offer a complementary perspective on region-specific treatment effects that may not be captured by whole-brain coordinate-based meta-analytic approaches.

## Discussion

In the present whole-brain ALE meta-analysis, we examined treatment-related neural activation changes associated with psychotherapy and pharmacotherapy in ADs using task-based fMRI studies. The findings revealed distinct, yet partially convergent, patterns of neural modulation across treatment modalities. Such patterns are broadly consistent with neurocognitive models of anxiety that emphasise dysregulation within prefrontal–limbic systems involved in threat appraisal and emotion regulation (Etkin and Wager 2007; Warren et al. 2015). Importantly, all results should be interpreted as exploratory, given the limited number of eligible studies and the use of an uncorrected statistical threshold.

The complementary behavioural synthesis showed a large pooled reduction in anxiety symptoms, consistent with prior evidence supporting psychological and pharmacological treatment in anxiety disorders (Hofmann and Smits 2008; Bandelow et al. 2012, 2015; Hoge et al. 2023). However, substantial between-cohort heterogeneity and assumed pre-post correlations warrant caution in interpreting the magnitude of this effect (Morris 2000; Cuijpers et al. 2017). Importantly, this behavioural improvement provides clinical context for the ALE findings but does not establish that a specific activation cluster mediates symptom change.

Age and sex were therefore retained as study-level descriptors rather than pooled estimates because reporting was incomplete and several publications drew on overlapping cohorts. Formal demographic pooling under these conditions could double-count participants and produce misleadingly precise estimates.

Across all 21 psychotherapy studies, treatment-related effects were predominantly characterised by decreased activation in prefrontal and striatal regions, including the medial, middle, and inferior frontal gyri as well as the caudate nucleus. These regions play a central role in cognitive control, self-referential processing, and habitual or threat-related responding, functions that are frequently altered in ADs (Etkin and Wager 2007). Rather than indicating reduced regulatory capacity, the observed decreases may reflect an attenuation or normalisation of heightened prefrontal–striatal engagement following psychotherapy. Similar interpretations have been proposed in prior neuroimaging meta-analyses of psychological interventions, suggesting that symptom improvement may be accompanied by reduced reliance on effortful control mechanisms (Messina et al. 2013).

When analyses were restricted to CBT, a more differentiated pattern emerged, with both increased and decreased activation observed across distinct regions. Increased activation in the ACG, IFG, and PG may reflect active engagement of emotion-processing and regulatory systems during exposure or cognitive restructuring, processes that are central to CBT-based interventions (Picó-Pérez et al. 2023). In contrast, decreased activation in lateral prefrontal regions and the caudate nucleus may indicate reduced reliance on effortful cognitive control and habitual response mechanisms as symptoms improve, a pattern that has also been reported in longitudinal neuroimaging studies of CBT (Bomyea et al. 2020). Together, these findings suggest that CBT-related neural changes involve region-specific modulation rather than uniform enhancement of control networks.

Psychotherapy-related neural modulation varied across diagnostic subgroups. In social anxiety disorder and panic disorder, psychotherapy was associated with decreased activation in prefrontal regions, with additional insular involvement observed in PD. These regions are implicated in threat appraisal and interoceptive monitoring, processes that are commonly heightened in these conditions (Freitas-Ferrari et al. 2010). In contrast, patients with SP exhibited increased activation in the ACG occipital cortices and IFG. This pattern may reflect enhanced processing of phobia-relevant stimuli during exposure-based interventions, consistent with prior evidence highlighting the role of visual and cingulate regions in fear-related stimulus processing (Binelli et al. 2014). Given the limited number of studies within each diagnostic subgroup, these interpretations should be considered tentative.

Task-based subgroup analyses focusing on emotional face paradigms further indicated increased activation in frontal and cingulate regions following psychotherapy. Emotional face processing has been shown to robustly engage prefrontal and cingulate systems involved in emotion regulation and social threat evaluation (Binelli et al. 2014; Gentili et al. 2016). Increased activation in these regions following psychotherapy may therefore reflect enhanced engagement of regulatory processes during affective challenges. As with other subgroup findings, these results should be interpreted cautiously.

With respect to pharmacotherapy, the ALE results indicated a more spatially restricted pattern of neural change, with a single cluster of decreased activation in the right uncus, a limbic region implicated in emotional and memory-related processing. This focal pattern is broadly consistent with pharmacological models proposing that anxiolytic medications primarily modulate bottom-up affective circuits, particularly within limbic structures, rather than inducing widespread changes in prefrontal control systems (Warren et al. 2015). Previous neuroimaging studies have similarly suggested that serotonergic antidepressants can alter emotional processing and limbic reactivity early in treatment, potentially preceding broader cognitive changes (Godlewska et al. 2012).

Taken together, the present findings suggest that psychotherapy and pharmacotherapy may engage partially distinct patterns of neural modulation in ADs. Prior comparative reviews of neuroimaging studies have similarly highlighted that psychological and pharmacological treatments can show differentiated neural effects, often interpreted as functional normalisation within fear-related circuits rather than identical neural change profiles across modalities (Quidé et al. 2012). From the perspective of psychological therapy mechanisms, meta-analytic work has suggested that psychotherapy-related changes may involve modulation of distributed regulatory systems and can be framed within dual-process accounts of treatment action (Marwood et al. 2018). In parallel, cognitive neuropsychological models of antidepressant action propose that pharmacotherapy may alter bottom-up affective processing biases and limbic reactivity early in treatment, with downstream consequences for emotion regulation and symptom improvement (Pringle et al. 2011). However, given the exploratory nature of the present coordinate-based analyses and the absence of formal cross-modality contrast or conjunction testing, any modality-level comparisons should be interpreted cautiously. A formal contrast ALE (e.g., psychotherapy > pharmacotherapy) was not performed, as the markedly imbalanced number of experiments and limited overlap between modalities would violate the statistical assumptions required for permutation-based inference.

The ROI-based observations summarised in the Results broadly complement the whole-brain findings by implicating limbic and paralimbic regions in treatment response. Nevertheless, because ROI analyses are hypothesis constrained and do not test whole-brain spatial convergence, they should be interpreted as supportive regional evidence rather than as direct confirmation of the ALE clusters.

Several limitations should be acknowledged. The limited number of eligible studies, particularly for pharmacotherapy and diagnostic subgroups, reduced statistical power and necessitated the use of an uncorrected statistical threshold. Substantial heterogeneity across task paradigms, treatment protocols, and imaging methods may also have influenced the observed convergence patterns. Future studies would benefit from larger, harmonised datasets, investigation of combined treatment approaches, multimodal imaging designs, and longitudinal assessments to clarify how neural modulation unfolds across treatment stages.

## Conclusions

This ALE meta-analysis provides a systematic, whole-brain overview of treatment-related neural activation changes associated with psychotherapy and pharmacotherapy in ADs. Across task-based fMRI studies, psychotherapy was predominantly associated with modulation of prefrontal and striatal regions, with additional heterogeneity observed across treatment modality, diagnostic category, and task paradigm. In contrast, pharmacotherapy-related neural changes were more spatially restricted and primarily involved limbic regions.

Importantly, these findings should be interpreted as exploratory, given the limited number of eligible studies, subgroup-specific sample sizes, and the use of an uncorrected statistical threshold. Rather than demonstrating definitive neural mechanisms, the present results highlight convergent and divergent patterns of neural modulation that may inform hypotheses for future research. Larger, methodologically harmonised, and longitudinal neuroimaging studies, particularly those examining combined treatments and multimodal imaging measures, will be essential to clarify how different interventions shape neural processes underlying ADs.

## Supplementary Material

Supplementary materials.docx
