# Supplementary material for: Prefrontal-limbic circuit modulation in anxiety disorders: Differential neural effects of psychotherapy versus pharmacotherapy revealed by ALE meta-analysis
Source: Dialogues Clin Neurosci. 2026 Aug 25;28(1):289–301. doi: 10.1080/19585969.2026.2719522 (PMC13520848; doi:10.1080/19585969.2026.2719522)
Supplement: Supplementary file 1 [file TDCN_A_2719522_SM7193.docx]

**Supplementary Table 1.** Search strategy for PubMed

| Search number | Query | Results |
| --- | --- | --- |
| 14 | ((("Anxiety Disorders"[Mesh] AND (2000/1/1:2025/12/31[pdat])) OR ((((((((((Anxiety Disorder) OR (Disorder, Anxiety)) OR (Disorders, Anxiety)) OR (Neuroses, Anxiety)) OR (Anxiety Neuroses)) OR (Anxiety States, Neurotic)) OR (Anxiety State, Neurotic)) OR (Neurotic Anxiety States)) OR (State, Neurotic Anxiety)) OR (States, Neurotic Anxiety) AND (2000/1/1:2025/12/31[pdat])) AND (2000/1/1:2025/12/31[pdat])) AND (("Drug Therapy"[Mesh] AND (2000/1/1:2025/12/31[pdat])) OR (((((((Chemotherapy) OR (Chemotherapies)) OR (Pharmacotherapy)) OR (Pharmacotherapies)) OR (Therapy, Drug)) OR (Drug Therapies)) OR (Therapies, Drug) AND (2000/1/1:2025/12/31[pdat])) AND (2000/1/1:2025/12/31[pdat]))) AND (("Magnetic Resonance Imaging"[Mesh] AND (2000/1/1:2025/12/31[pdat])) OR ((((((((fMRI) OR (Magnetic Resonance Imaging, Functional)) OR (MRI, Functional)) OR (Functional MRI)) OR (Functional MRIs)) OR (MRIs, Functional)) OR (Functional Magnetic)) OR (Resonance Imaging) AND (2000/1/1:2025/12/31[pdat])) AND (2000/1/1:2025/12/31[pdat])) | 742 |
| 13 | ((("Anxiety Disorders"[Mesh] AND (2000/1/1:2025/12/31[pdat])) OR ((((((((((Anxiety Disorder) OR (Disorder, Anxiety)) OR (Disorders, Anxiety)) OR (Neuroses, Anxiety)) OR (Anxiety Neuroses)) OR (Anxiety States, Neurotic)) OR (Anxiety State, Neurotic)) OR (Neurotic Anxiety States)) OR (State, Neurotic Anxiety)) OR (States, Neurotic Anxiety) AND (2000/1/1:2025/12/31[pdat])) AND (2000/1/1:2025/12/31[pdat])) AND (("Psychotherapy"[Mesh] AND (2000/1/1:2025/12/31[pdat])) OR (Psychotherapies AND (2000/1/1:2025/12/31[pdat])) AND (2000/1/1:2025/12/31[pdat]))) AND (("Magnetic Resonance Imaging"[Mesh] AND (2000/1/1:2025/12/31[pdat])) OR ((((((((fMRI) OR (Magnetic Resonance Imaging, Functional)) OR (MRI, Functional)) OR (Functional MRI)) OR (Functional MRIs)) OR (MRIs, Functional)) OR (Functional Magnetic)) OR (Resonance Imaging) AND (2000/1/1:2025/12/31[pdat])) AND (2000/1/1:2025/12/31[pdat])) | 882 |
| 12 | ("Magnetic Resonance Imaging"[Mesh] AND (2000/1/1:2025/12/31[pdat])) OR ((((((((fMRI) OR (Magnetic Resonance Imaging, Functional)) OR (MRI, Functional)) OR (Functional MRI)) OR (Functional MRIs)) OR (MRIs, Functional)) OR (Functional Magnetic)) OR (Resonance Imaging) AND (2000/1/1:2025/12/31[pdat])) | 875,267 |
| 11 | (((((((fMRI) OR (Magnetic Resonance Imaging, Functional)) OR (MRI, Functional)) OR (Functional MRI)) OR (Functional MRIs)) OR (MRIs, Functional)) OR (Functional Magnetic)) OR (Resonance Imaging) | 875,267 |
| 10 | "Magnetic Resonance Imaging"[Mesh] | 515,936 |
| 9 | ("Drug Therapy"[Mesh] AND (2000/1/1:2025/12/31[pdat])) OR (((((((Chemotherapy) OR (Chemotherapies)) OR (Pharmacotherapy)) OR (Pharmacotherapies)) OR (Therapy, Drug)) OR (Drug Therapies)) OR (Therapies, Drug) AND (2000/1/1:2025/12/31[pdat])) | 3,195,105 |
| 8 | ((((((Chemotherapy) OR (Chemotherapies)) OR (Pharmacotherapy)) OR (Pharmacotherapies)) OR (Therapy, Drug)) OR (Drug Therapies)) OR (Therapies, Drug) | 3,195,105 |
| 7 | "Drug Therapy"[Mesh] | 1,083,300 |
| 6 | ("Psychotherapy"[Mesh] AND (2000/1/1:2025/12/31[pdat])) OR (Psychotherapies AND (2000/1/1:2025/12/31[pdat])) | 203,219 |
| 5 | Psychotherapies | 203,219 |
| 4 | "Psychotherapy"[Mesh] | 141,166 |
| 3 | ("Anxiety Disorders"[Mesh] AND (2000/1/1:2025/12/31[pdat])) OR ((((((((((Anxiety Disorder) OR (Disorder, Anxiety)) OR (Disorders, Anxiety)) OR (Neuroses, Anxiety)) OR (Anxiety Neuroses)) OR (Anxiety States, Neurotic)) OR (Anxiety State, Neurotic)) OR (Neurotic Anxiety States)) OR (State, Neurotic Anxiety)) OR (States, Neurotic Anxiety) AND (2000/1/1:2025/12/31[pdat])) | 174,160 |
| 2 | (((((((((Anxiety Disorder) OR (Disorder, Anxiety)) OR (Disorders, Anxiety)) OR (Neuroses, Anxiety)) OR (Anxiety Neuroses)) OR (Anxiety States, Neurotic)) OR (Anxiety State, Neurotic)) OR (Neurotic Anxiety States)) OR (State, Neurotic Anxiety)) OR (States, Neurotic Anxiety) | 174,160 |
| 1 | "Anxiety Disorders"[Mesh] | 59,267 |

**Supplementary Table 2.** Quality assessment checklist (total score out of 10)

**Criteria 1: Participants**

1. Patients were prospectively assessed; specific diagnostic criteria and demographic data were reported.

2. Healthy controls were prospectively evaluated; psychiatric and medical illnesses were excluded.

3. Important variables (e.g., age, sex, illness duration, onset, medication status, comorbidity, and the severity of illness) were reported.

4. Sample size per group $\geq$ 10.

**Criteria 2: Methods for image acquisition and analysis**

5. Whole-brain analysis was automated with no a priori regional selection.

6. Coordinates reported in a standard space.

7. The imaging technique used was clearly described so that it could be reproduced.

8. Measurements were clearly described so that they could be reproduced.

**Criteria 3: Results and conclusions**

9. Statistical parameters for significant nonsignificant differences were provided.

10. Conclusions were consistent with the results obtained and the limitations were discussed.

Didn’t match the criteria, 0 points were awarded;

Partially met the criteria, 0.5 points were awarded;

Fully met the criteria, 1 point was awarded.

**Supplementary Table 3.** Quality assessment of included studies

| Study ID | Criteria 1 | Criteria 2 | Criteria 3 | Quality |
| --- | --- | --- | --- | --- |
|  | (Max 4 points) | (Max 4 points) | (Max 2 points) |  |
| Brown L A | 4 | 3 | 2 | 9 |
| Goldin P R2009 | 4 | 2 | 2 | 8 |
| Goldin P R2012 | 3.5 | 4 | 2 | 9.5 |
| Hauner K K | 4 | 4 | 2 | 10 |
| Hölzel B K | 4 | 4 | 2 | 10 |
| Klumpp H2013 | 4 | 3 | 1.5 | 8.5 |
| Klumpp H2016 | 3 | 4 | 2 | 9 |
| Klumpp H2014 | 3 | 4 | 2 | 9 |
| Kunas S L | 3 | 3.5 | 2 | 8.5 |
| Lipka J | 4 | 3.5 | 2 | 9.5 |
| Lueken U | 3.5 | 4 | 2 | 9.5 |
| Månsson K N T 2013 | 3.5 | 4 | 2 | 9.5 |
| Kircher | 4 | 3.5 | 1.5 | 9 |
| Yolanda | 4 | 3.5 | 2 | 9.5 |
| Ji-Won Hur | 3.5 | 4 | 2 | 9.5 |
| Furmark | 4 | 4 | 2 | 10 |
| Kim | 4 | 3 | 2 | 9 |
| Azriel | 3.5 | 4 | 2 | 9.5 |
| Bomyea | 4 | 4 | 2 | 10 |
| Schienle | 3 | 4 | 2 | 9 |
| Straube T | 3.5 | 4 | 2 | 9.5 |
| Schneier F R | 3.5 | 3 | 2 | 8.5 |
| Phan K L | 3 | 3 | 2 | 8 |
| Hyun-Bo | 4 | 3.5 | 2 | 9.5 |
| Kilts D | 4 | 3 | 2 | 9 |
| Faria | 3 | 4 | 2 | 9 |
| Hoehn-Saric R | 4 | 4 | 2 | 10 |

Quality is the sum of Criteria 1-3. Domain scores were constrained to their stated maxima of 4, 4, and 2 points, respectively, and total scores were recalculated from the displayed domain scores.

**Supplementary Table 4.** Results of ALE meta-analysis activation clusters in patients with ADs treated with CBT

| Hemisphere | BA | Effect lobe | Effect gyrus | Center coordinates | | | Volume/mm³ | ALE value* |
| --- | --- | --- | --- | --- | --- | --- | --- | --- |
|  |  |  |  | X | Y | Z |  |  |
| Increase： |  |  |  |  |  |  |  |  |
| right | 32 | Limbic Lobe | PG | 40 | -18 | -18 | 456 | 0.008 |
| right | 32 | Limbic Lobe | ACG | 8 | 36 | -14 | 456 | 0.008 |
| Left | 47 | Limbic Lobe | ACG | -12 | 34 | -12 | 360 | 0.007 |
| right |  | Frontal Lobe | IFG | 18 | 30 | -26 | 328 | 0.007 |
| Decrease: |  |  |  |  |  |  |  |  |
| left | 46 | Frontal Lobe | MFG | -50 | 36 | 10 | 648 | 0.017 |
| left | 44 | Frontal Lobe | IFG | -50 | 10 | 14 | 512 | 0.013 |
| right |  | Sub-lobar | Caudate | 12 | 8 | 12 | 280 | 0.010 |

*The results are in scientific notation and are actually ALE value x 10^-2^. The peak coordinates were in the MNI system.Uncorrected p<0.001, mni.cluster size=250mm^3^ ACG: Anterior Cingulate Gyrus; ALE: Activation Likelihood Estimation; BA: Brodmann Area; CBT: Cognitive behavioral therapy; IFG: Inferior Frontal Gyrus; MFG: Middle Frontal Gyrus; PG: Parahippocampal Gyrus.

**Supplementary Table 5.** Results of ALE meta-analysis activation clusters in patients with SAD treated with psychotherapy

| Hemisphere | BA | Effect lobe | Effect gyrus | Center coordinates | | | Volume/mm³ | ALE value* |
| --- | --- | --- | --- | --- | --- | --- | --- | --- |
|  |  |  |  | X | Y | Z |  |  |
| Decrease： |  |  |  |  |  |  |  |  |
| left | 8 | Frontal Lobe | MeFG | -2 | 36 | 40 | 360 | 0.010 |

*The results are in scientific notation and are actually ALE value x 10^-2^. The peak coordinates were in the MNI system. Uncorrected p<0.001, mni.cluster size=250mm3. ALE: Activation Likelihood Estimation; BA: Brodmann Area; MeFG: Medial Frontal Gyrus.

**Supplementary Table 6.** Results of ALE meta-analysis activation clusters in patients with PD treated with psychotherapy

| Hemisphere | BA | Effect lobe | Effect gyrus | Center coordinates | | | Volume/mm³ | ALE value* |
| --- | --- | --- | --- | --- | --- | --- | --- | --- |
|  |  |  |  | X | Y | Z |  |  |
| Decrease： |  |  |  |  |  |  |  |  |
| left | 46 | Frontal Lobe | MFG | -50 | 36 | 10 | 763 | 0.017 |
| left | 13 | Sub-lobar | Insula | -48 | 11 | 14 | 384 | 0.008 |

*The results are in scientific notation and are actually ALE value x 10^-2^. The peak coordinates were in the MNI system. Uncorrected p<0.001, mni.cluster size=250mm3. ALE: Activation Likelihood Estimation; BA: Brodmann Area; MFG: Middle Frontal Gyrus.

**Supplementary Table 7.** Results of ALE meta-analysis activation clusters in patients with SP treated with psychotherapy

| Hemisphere | BA | Effect lobe | Effect gyrus | Center coordinates | | | Volume/mm³ | ALE value* |
| --- | --- | --- | --- | --- | --- | --- | --- | --- |
|  |  |  |  | X | Y | Z |  |  |
| Increase： |  |  |  |  |  |  |  |  |
| left | 32 | Limbic Lobe | ACG | -12 | 33 | -12 | 480 | 0.007 |
| left | 18 | Occipital Lobe | MOG | -36 | -86 | -6 | 480 | 0.007 |
| right | 19 | Occipital Lobe | FG | 40 | -82 | -10 | 456 | 0.007 |
| right | 47 | Frontal Lobe | IFG | 18 | 30 | -27 | 416 | 0.007 |

*The results are in scientific notation and are actually ALE value x 10^-2^. The peak coordinates were in the MNI system. Uncorrected p<0.001, mni.cluster size=250mm3. ACG: Anterior Cingulate Gyrus; ALE: Activation Likelihood Estimation; BA: Brodmann Area; IFG: Inferior Frontal Gyrus; MOG: Middle Occipital Gyrus; FG: Frontal Gyrus.

**Supplementary Table 8.** Results of ALE meta-analysis activation clusters in patients with ADs who use emotional face tasks after psychotherapy

| Hemisphere | BA | Effect lobe | Effect gyrus | Center coordinates | | | Volume/mm³ | ALE value* |
| --- | --- | --- | --- | --- | --- | --- | --- | --- |
|  |  |  |  | X | Y | Z |  |  |
| Increase |  |  |  |  |  |  |  |  |
| right | 45 | Frontal Lobe | IFG | 52 | 28 | 4 | 264 | 0.009 |
| left | 24 | Limbic Lobe | ACG | 2 | 26 | 16 | 264 | 0.009 |
| right | 7 | Parietal Lobe | PreCG | 9 | -49 | 45 | 256 | 0.007 |

*The results are in scientific notation and are actually ALE value x 10^-2^. The peak coordinates were in the MNI system.Uncorrected p<0.001, mni.cluster size=250mm^3^. ACG: Anterior Cingulate Gyrus; ALE: Activation Likelihood Estimation; BA: Brodmann Area; IFG: Inferior Frontal Gyrus; PreCG: Precentral Gyrus

**Supplementary Table 9.** Narrative overview of region-of-interest (ROI) findings from the included fMRI studies

| Study | Disorder | Treatment | ROI region | Direction |
| --- | --- | --- | --- | --- |
|  |  |  |  |  |
| Straube T | SP | CBT | AC; Insula;PG;Thalamus | ↓ |
| Lipka J | SP | CBT | Amygdala; PdACC | ↓ |
| Månsson K N T 2013 | SAD | CBT | Left Amygdala | ↓ |
| Månsson K N T 2013 | SAD | ABM | Amygdala | ↑ |

ABM: Attentional Bias Modification Therapy; ACG: Anterior Cingulate Gyrus; CBT:Cognitive Behavioral Therapy; PdACG: posterior dorsal Anterior Cingulate Gyrus;PG: Parahippocampal Gyrus; SAD: social anxiety disorder; SP: Spider Phobia.

**Supplementary Table 10.** Cohort-level inputs and effect sizes for the behavioral synthesis

| Study/cohort | Treatment | Measure | N | Pre M (SD) | Post M (SD) | Hedges' g | SE (g) |
| --- | --- | --- | --- | --- | --- | --- | --- |
| Brown et al. [29], CBT | CBT | LSAS | 17 | 81.55 (15.75) | 61.82 (20.47) | 1.01 | 0.289 |
| Brown et al. [29], ACT | ACT | LSAS | 20 | 90.92 (21.25) | 66.75 (19.98) | 1.12 | 0.279 |
| Goldin et al. [30] | MBSR | LSAS | 14 | 68.70 (21.20) | 49.30 (17.00) | 0.94 | 0.308 |
| Hur et al. [43] | VR psychotherapy | SPS | 21 | 30.86 (13.34) | 23.52 (12.34) | 0.55 | 0.226 |
| Bomyea et al. [47] | CBT | OASIS | 30 | 8.50 (3.40) | 3.67 (3.38) | 1.39 | 0.252 |
| Faria et al. [54], overt | Escitalopram | LSAS-SR | 24 | 83.71 (20.13) | 41.08 (17.91) | 2.11 | 0.363 |
| Faria et al. [54], covert | Escitalopram | LSAS-SR | 22 | 81.45 (17.82) | 60.00 (20.17) | 1.35 | 0.289 |
| Alvarez-Perez et al. [42], in vivo | Exposure | S-R | 17 | 37.94 (6.75) | 18.60 (6.63) | 2.75 | 0.526 |
| Alvarez-Perez et al. [42], VRET | VRET | S-R | 14 | 37.86 (5.76) | 17.21 (8.11) | 2.69 | 0.567 |
| Brown et al. [29], CBT | CBT | LSAS | 17 | 81.55 (15.75) | 61.82 (20.47) | 1.01 | 0.289 |

One primary anxiety measure was selected per independent active-treatment cohort. Positive Hedges' g values denote symptom reduction. For cohorts without a reported standard deviation of change, the primary calculation assumed a pre-post correlation of r = 0.50; Faria et al. reported the standard deviation of change. ACT: acceptance and commitment therapy; CBT: cognitive behavioral therapy; LSAS: Liebowitz Social Anxiety Scale; LSAS-SR: Liebowitz Social Anxiety Scale-Self-Report; MBSR: mindfulness-based stress reduction; OASIS: Overall Anxiety Severity and Impairment Scale; SPS: Social Phobia Scale; VRET: virtual reality exposure therapy. SE (g) is the cohort-level standard error used for inverse-variance weighting in the random-effects model; a normal-approximation 95% confidence interval can be calculated as g ± 1.96 × SE.

 **Supplementary Fig. 1** ALE meta-analysis of activation cluster results in patients with ADs treated with CBT. Uncorrected p<0.001, mni.cluster size=250mm3. The brain regions activated in the Fig. reached a significant activation level, with areas of increased activity in red and areas of decreased activity in blue. The diagrams from left to right are: right Parahippocampal Gyrus; left Anterior Cingulate Gyru; left Anterior Cingulate Gyru; right Inferior Frontal Gyrus; left Middle Frontal Gyrus; left Inferior Frontal Gyrus; right Caudate.

**Supplementary Fig. 2** ALE meta-analysis of activation cluster results in patients with SAD treated with psychotherapy. Uncorrected p<0.001, mni.cluster size=250mm3. The brain regions activated in the Fig. reached a significant activation level, with areas of increased activity in red and areas of decreased activity in blue. The diagrams from left to right are: left Medial Frontal Gyrus.

**Supplementary Fig. 3** ALE meta-analysis of activation cluster results in patients with PD treated with psychotherapy. Uncorrected p<0.001, mni.cluster size=250mm3. The brain regions activated in the Fig. reached a significant activation level, with areas of increased activity in red and areas of decreased activity in blue. The diagrams from left to right are: left Middle Frontal Gyrus; left Insula.

**Supplementary Fig. 4** ALE meta-analysis of activation cluster results in patients with SP treated with psychotherapy. Uncorrected p<0.001, mni.cluster size=250mm3. The brain regions activated in the Fig. reached a significant activation level, with areas of increased activity in red and areas of decreased activity in blue. The diagrams from left to right are: right Anterior Cingulate Gyrus; left Middle Occipital Gyrus; right Frontal Gyrus; left Inferior Frontal Gyrus.

**Supplementary Fig. 5** Graph of activation cluster results using the emotional faces task ALE meta-analysis in patients with ADs following psychotherapy. Uncorrected p<0.001, mni.cluster size=250mm3. The brain regions activated in the Fig. reached a significant activation level, with areas of increased activity in red and areas of decreased activity in blue. The diagrams from left to right are: right Inferior Frontal Gyrus; right Precentral Gyrus; left Anterior Cingulate Gyrus.

 **Supplementary Fig. 6.** Forest plot of within-cohort standardized mean anxiety symptom reduction under the primary assumption (r = 0.50). Squares denote cohort-level Hedges' g estimates, horizontal lines denote 95% confidence intervals, and the diamond denotes the random-effects REML pooled estimate. Positive values indicate symptom reduction.

**Supplementary Fig. 7.** Sensitivity analysis of the pooled standardized mean symptom reduction across assumed pre-post correlations of r = 0.30, 0.50, and 0.70. Points denote pooled Hedges' g estimates and error bars denote 95% confidence intervals.
